# Cigarette Smoke Extract Promotes Human Vascular Smooth Muscle Cell Proliferation and Survival through ERK1/2- and NF-κB–Dependent Pathways

**DOI:** 10.1100/tsw.2010.201

**Published:** 2010-11-04

**Authors:** Qing-wen Chen, Lars Edvinsson, Cang-Bao Xu

**Affiliations:** ^1^ Division of Experimental Vascular Research, Institute of Clinical Science in Lund, Lund University, Lund, Sweden; ^2^ Department of Clinical and Experimental Research, Glostrup Research Center, Glostrup Hospital, Copenhagen University, Copenhagen, Denmark

**Keywords:** smoke particles, human vascular smooth muscle cells, cell proliferation, cell death, ERK1/2, NF-kappaB

## Abstract

Tobacco use is one of the major risk factors of cardiovascular disease. The underlying molecular mechanisms that link cigarette smoke to cardiovascular disease remain unclear. The present study was designed to examine the effects of dimethyl sulfoxide (DMSO)–soluble smoke particles (DSPs) on human aortic smooth muscle cell (HASMC) cultures, and to explore the mitogen-activated protein kinase (MAPK)/extracellular signal-regulated protein kinase 1 and 2 (ERK1/2) and nuclear factor-κB (NF-κB) signal mechanisms involved. Serum-starved HASMCs were treated with DSPs for up to 48 h. DSPs promoted cell proliferation in a concentration-dependent manner from 0.05 to 0.2 ?l/ml. Activation of ERK1/2 and NF-κB was seen after exposure to DSPs. This occurred in parallel with the increase in cell population, bromodeoxyuridine incorporation, and cyclinD1/cyclin-dependent kinase 4 expression. Blocking phosphorylation of ERK1/2 by MAPK inhibitors U0126 and PD98059, and inhibiting activation of NF-κB by IκB (IκB) kinase inhibitors wedelolactone or IMD-0354, abolished the DSP effects. However, either a p38 inhibitor (SB203580) or an inhibitor of lipopolysaccharide (polymyxin B), or nicotinic receptor blockers (mecamylamine and α-bungarotoxin), did not inhibit a DSP-induced increase in the cell population. DSPs increased the expression of intercellular adhesion molecule 1 and the release of interleukin-6 in HASMCs, both of which were inhibited by ERK1/2 or NF-κB pathway inhibitors. Furthermore, cell apoptosis and necrosis were found in serum-starved HASMCs. DSPs decreased cell death and increased B-cell leukemia/lymphoma 2 expression. Blocking phosphorylation of ERK1/2 or NF-κB attenuated DSP-induced cell death inhibition. Cigarette smoke particles stimulate HASMC proliferation and inhibit cell death. The intracellular signal mechanisms behind this involve activation of ERK1/2 and NF-κB pathways.

### INTRODUCTION

The pathogenesis of atherosclerosis and restenosis is characterized by accumulation of smooth muscle cells within the neointima. The homeostatic balance between vascular smooth muscle cell (VSMC) growth and cell death is pivotal for vascular remodeling, plaque formation, and its vulnerability[1,2,3]. It is hypothesized that stimulation of VSMC proliferation and inhibition of VSMC apoptosis may be necessary for plaque formation[1]. An excessive and chronic inflammatory response of the vascular wall to various forms of stimuli and injury, such as cytokines, growth factors, cigarette smoke, hypertension, hyperlipidemia, diabetes, and inflammation, is involved in the formation and development of atherosclerosis[4,5,6].

Cigarette smoke is one of several well-recognized risk factors for vascular inflammation and associated cardiovascular diseases, such as coronary heart disease, stroke, and myocardial infarction[7,8,9]. However, the mechanisms that are responsible for cigarette smoke leading to cardiovascular disease are still poorly understood. Cigarette smoke is a complex chemical mixture that contains over 4000 different compounds, in which 158 of the chemical constituents are identified to be toxic hazards, 17 of noncancer compounds (based on a noncancer risk index), and 15 of individual chemical constituents contribute to cardiovascular dysfunction[10]. Previous studies suggest that cigarette smoke may induce vascular remodeling through complicated intracellular signal mechanisms[11,12]. Extracellular signal-regulated protein kinase 1 and 2 (ERK1/2), which is one of the members of the mitogen-activated protein kinase (MAPK) superfamily, acts as an important regulator of cellular proliferation, migration, and differentiation[13,14]. Activation of the downstream transcriptional factor NF-kappaB (NF-κB) regulates gene expression, promotes cell survival, and protects cells from apoptosis[15,16,17,18,19,20]. Our previous studies showed that both lipid- and water-soluble cigarette smoke extracts promoted cell proliferation in human aortic smooth muscle cells (HASMCs), but the effect of lipid-soluble smoke extracts occurred earlier. Furthermore, lipid-soluble, but not water-soluble smoke extracts increased the G protein-coupled receptor (GPCR)–mediated contractile responses in rat vasculature and airways[21]. Dimethyl sulfoxide (DMSO)–soluble smoke particles (DSPs) include both lipid- and water-soluble particles from cigarette smoke. The present study was designed to examine the hypothesis that DSPs may induce inflammatory response and enhance human VSMC cell survival through activation of the ERK1/2 and NF-κB pathways. Understanding the intracellular signal mechanisms that leads cigarette smoke to cardiovascular disease may provide novel options for treatment.

### MATERIALS AND METHODS

#### Extraction of Cigarette Smoke Particles

For the preparation method of cigarette smoke particles, please refer to Granström et al.[22]. Three cigarettes (Marlboro, 0.8-mg nicotine per cigarette) were “smoked” by an aspirator and the smoke was collected through a cotton wool filter. The retained smoke in the filter was dissolved in 1 ml of DMSO to produce DSPs. The DSP preparations were analyzed by gas chromatography and then diluted by DMSO to standard nicotine content (0.1 mg/ml)[22]. These stock solutions were kept at -20°C and used for the experiments within 3 months. DMSO was used as a vehicle control in all experiments.

#### Cell Culture

HASMCs at the end of the tertiary culture stage were obtained as a commercially available product from Cascade Biologics (Portland, OR). Cells were plated in 75-cm$^2$ tissue culture flasks at a density of 2.5 × 10$^3$ viable cells/cm$^2$ in Medium 231 supplemented with 5% smooth muscle growth supplement (SMGS). Medium 231 and SMGS were purchased from Cascade Biologics. The cells were incubated in a 5% $CO_2$

incubator at 37°C and the medium was replaced every other day until the culture was approximately 80–90% confluent. Then the cells were removed from the flasks with Accutase™ Enzyme Cell Detachment Medium (eBioscience, San Diego, CA) and seeded onto a 100-mm tissue culture dish (Greiner Bio-One, Frickenhausen, Germany). All experiments were performed with the cells from passages 6 to 9.

### Cell Proliferation and Cytotoxicity Assay

HASMCs were seeded at a density of $4 \times 10^3$ per well in a 96-well plate for 1 day and then starved in Medium 231 with 0.05% SMGS for 24 h. Different concentrations of DSPs or vehicle DMSO were added to the cells for 48 h. After 1 h of DSP or vehicle treatment, the cells were incubated in the absence or the presence of 10 nM of endothelin-1 (ET-1) for 48 h. The inhibitors were added to the cells 30 min prior to the addition of DSPs. The cell population in the 96-well plate were analyzed by a nonradioactive cell proliferation and cytotoxicity assay kit (EZ4U kit, BIOMEDICA GRUPPE, Vienna, Austria) following the manufacturer's instructions. Independent experiments were done in triplicate and were repeated at least three times.

### Bromodeoxyuridine Incorporation Enzyme-Linked Immunosorbent Assay (ELISA)

Cell proliferation was identified with the incorporation of bromodeoxyuridine (BrdU) into cellular DNA. HASMCs were seeded in a 96-well plate and serum starved for 24 h. The cells were treated with 0.2 µl/ml of DSPs for 24 h and the inhibitors were added to the cells 30 min prior to the addition of DSPs. At 2 h before the end of the incubation, BrdU labeling solution was added to the cells and then Cell Proliferation ELISA (Roche Applied Science, Mannheim, Germany) was used to determine BrdU incorporation following the manufacturer's instructions.

### Cell Apoptosis/Necrosis Assay

HASMCs were seeded in a 96-well plate and serum starved for 24 h. The cells were treated with 0.2 µl/ml of DSPs for 48 h and inhibitors were added to the cells 30 min prior to the addition of DSPs. Cell apoptosis and necrosis were determined immediately by Cell Death Detection ELISA (Roche Applied Science) following the manufacturer's instructions. The cells from the same population that were incubated in the culture medium with complete serum were regarded as negative control for this assay. The enrichment factors of DNA fragment released into the cytoplasm (apoptosis) and culture medium (necrosis) were calculated relative to the negative control.

### Cell-Based phosphoELISA Assay

HASMCs were seeded in a 96-well plate and serum starved for 24 h. The cells were treated with vehicle or different inhibitors for 30 min prior to the addition of DSPs. After 1, 2, or 3 h of treatment with DSPs at 0.2 µl/ml, the cells were fixed and stored at 4°C until the performance of experiments. Phosphorylated ERK1/2 or NF-κB was measured using a cell-based ELISA Assay Kit (SABiosciences, Frederick, MD) following the manufacturer's instructions. Phosphorylated ERK1/2 or NF-κB activity was presented as a relative extent to the level of total ERK1/2.

### Human Single Cytokine ELISA Assay

HASMCs were seeded in a 96-well plate and serum starved for 24 h. The cells were treated with 0.2 μl/ml of DSPs for 24 h and inhibitors were added to the cells 30 min prior to the addition of DSPs. The cell culture medium was collected and centrifuged for 10 min. The supernatants were aliquoted and stored at -20°C until the performance of experiments.

Using the "Human Multi-Analyte Profiler ELISArray kit" (SABiosciences) that includes 12 cytokines and chemokines (IL-1β, IL-4, IL-6, IL-10, IL-12, IL-17A, IFN-γ, TNF-α, TGF-β1, MCP1, MIP-1a, MIP-1b), we scanned the production of inflammatory mediators by DSP-treated HASMCs and found the level of interleukin-6 (IL-6) in the culture medium increased most significantly among all others. Therefore, IL-6 was chosen for further studies. The level of human IL-6 in cell culture supernatants was measured using a Single Analyte ELISArray kit (SABiosciences) following the manufacturer's instructions.

### Immunofluorescence Analysis

Immunofluorescence analysis has been described in detail previously[23]. Briefly, HASMCs were seeded in 4-well NUNC Lab-Tek II Chamber Slides and were serum starved for 24 h. After treatment with vehicle or DSPs, the cells were then washed, fixed, and permeabilized. The monoclonal or polyclonal primary antibodies were added to the cells, followed by adding fluorescein isothiocynate (FITC)–conjugated goat antimouse or antirabbit secondary antibody (1:1000-5000, Abcam, Cambridge, U.K.). In the control experiments, either the primary or secondary antibody was omitted. After washing with phosphate buffered saline (PBS), ProLong Gold antifade mounting reagent with 4',6-diamidino-2-phenylindole (DAPI) (Invitrogen, Carlsbad, CA) was added and the cells were sealed with cover slip on the slide. The immunofluorescence-stained cells were observed under a laser-scanning confocal microscope (Nikon, C1plus, Nikon Instruments, Melville, NY) and analyzed by ImageJ software (http://rsb.info.nih.gov/ij). The fluorescence intensity of cells was measured at four preset areas per sample and at least three independent experiments were performed. The fluorescence intensity of each treated group was determined as the percent increase over control, with the control normalized to 100%.

### Western Blot Analysis

Western blot analysis has been described in detail previously[23]. Briefly, about 70–80% confluent HASMCs were made quiescent by placing them in serum-free medium for 24 h and harvested in cell extract denaturing buffer (BioSource, Invitrogen) with addition of a protease inhibitor cocktail (Sigma, USA) after treatments. For phosphorylated protein extraction, a phosphatase inhibitor cocktail (Sigma, St. Louis, MO) was added to the cell extract solution. Whole cell lysates were sonicated and centrifuged. The supernatants were collected as protein samples. The protein concentrations were determined using the protein assay reagents (Bio-Rad, Hercules, CA) and stored at -80ºC until the assay. The protein homogenates were diluted 7:2:1(v/v) with 5× sodium dodecyl sulfate (SDS) sample buffer and 10× dithiothreitol (DTT) reducer (PAGE Gel, San Diego, CA). Of total proteins, 40–50 μg were boiled and separated by 4–12% SDS Ready Gels (PAGE Gel), and transferred electrophoretically to nitrocellulose membranes (Bio-Rad). The membrane was then blocked and incubated with primary antibodies, followed by incubation with horseradish peroxidase (HRP)–conjugated antimouse or antirabbit secondary antibodies (Amersham Biosciences, Piscataway, NJ). The probed proteins were developed by Immun-Star WesternC Chemiluminescent HRP Substrate kit (Bio-Rad). To detect multiple signals using a single membrane, the membrane was stripped with Restore PLUS Western Blot Stripping Buffer (Pierce Biotechnology, Rockford, IL). The membranes were visualized using a Fujifilm LAS-1000 Luminiscent Image Analyzer (Stamford, CT) and then quantification of band intensity was analyzed with Image Gauge Ver. 4.0 (Fuji Photo Film, Tokyo, Japan). Three independent experiments were performed in duplicate.

### Reagents and Antibodies

MAPK kinase 1 (MEK1) inhibitor PD98059 and MEK1/2 inhibitor U0126; IκB kinase inhibitors wedelolactone and IMD-0354; p38 inhibitor SB203580; nonselective NF-κB and activated protein-1 (AP-1) inhibitor dexamethasone; nicotinic receptor (AChR) blockers mecamylamine and α-bungarotoxin; lipopolysaccharide (LPS) inhibitor polymyxin B and ET-1 were purchased from Sigma-Aldrich (St. Louis, MO). The concentration of inhibitors was determined by recommendation from product data sheets and literature. ET-1 was dissolved in sterile water with 0.1% bovine serum albumin; the other reagents were dissolved in DMSO as a stock solution and diluted in cell culture medium before use.

Monoclonal antibodies for phosphorylated ERK1/2, intercellular adhesion molecule 1 (ICAM-1), cyclin-dependent kinase 4 (cdk4), and a polyclonal antibody for total ERK1/2 were obtained from Abcam (Cambridge, U.K.). Monoclonal antibodies for phosphorylated NF-κB p56, total–NF-κB p56, cyclinD1, and polyclonal antibodies for phosphorylated p38, B-cell leukemia/lymphoma 2 (Bcl-2), and β-actin were purchased from Cell Signaling Technology (Boston, MA).

### Statistical Analysis

Comparison between two groups was performed using two-tailed unpaired Student's t-test with Welch's correction. For more than two groups, one-way ANOVA, followed by Dunnett's post-test was used. A $p$ value less than 0.05 was considered to be significant. Results were presented as mean ± SEM. At least three different samples or independent experiments were analyzed in each group.

## RESULTS

### DSPs Promote Cell Proliferation in a Concentration-Dependent Manner through Activation of ERK1/2 and NF-κB in HASMCs

Cell proliferation was first determined by a rapid and reliable, nonisotopic, cell proliferation assay. We found that DSPs caused an increase in cell population and increased ET-1–induced proliferation in a concentration-dependent manner from 0.05 to 0.2 μl/ml after 48 h of treatment compared with vehicle treatment (Fig. 1A). Both the cells cultured with and without ET-1 showed an increased cell proliferation from a lower (0.05 or 0.1 μl/ml) to a higher (0.1 or 0.2 μl/ml) concentration of DSPs ($p < 0.001$, $p < 0.05$). There was a significant difference between the cells cultured with and without ET-1 when the cells were treated with 0.05 μl/ml ($p < 0.01$) and 0.1 μl/ml ($p < 0.05$) of DSPs. Treatment with a higher concentration of DSPs (0.15 and 0.2 μl/ml) did not show a significant difference between these two groups. This may due to the limitations to cell growth under the serum starvation. The cell proliferation effect was diminished by pretreatment with MEK inhibitors PD98059 and U0126, NF-κB inhibitors wedelolactone and IMD-0354, and the nonselective NF-κB and AP-1 inhibitor dexamethasone, respectively, but not by the p38 inhibitor SB203580, the LPS inhibitor polymyxin B, or nicotinic AChR blockers mecamylamine and α-bungarotoxin (Fig. 1B). Cell proliferation was further verified by Cell Proliferation ELISA assay (BrdU incorporation). The DSP-induced increase in BrdU incorporation was prevented by U0126, wedelolactone, and IMD-0354, respectively (Fig. 1C).

### DSPs Induce Activation of ERK1/2, NF-κB, and p38 in HASMCs

To understand the involvement of activation of ERK1/2 and NF-κB in DSP-induced cell proliferation, we investigated the phosphorylation of ERK1/2 and NF-κB by immunofluorescence and western blot. After 2 h of treatment with DSPs, phosphorylated ERK1/2, NF-κB, and p38 were significantly increased in HASMCs

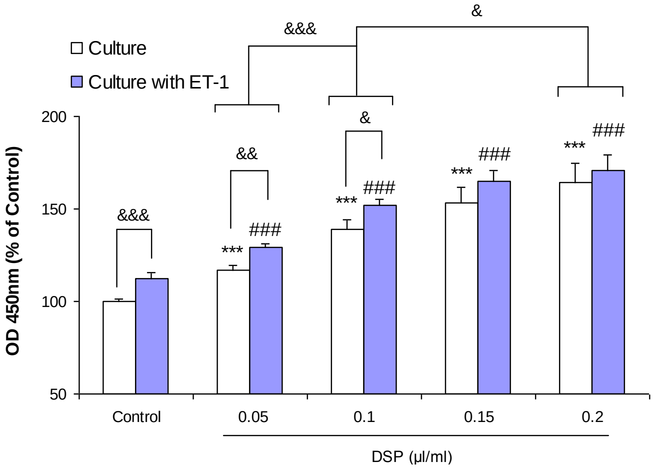

**A**

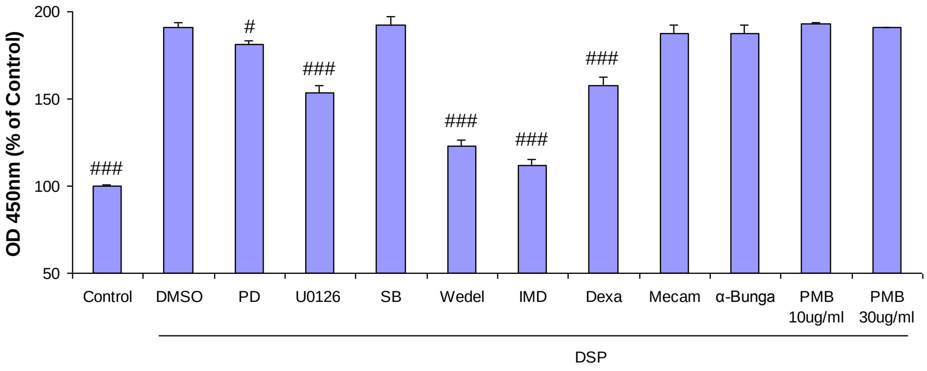

**B**

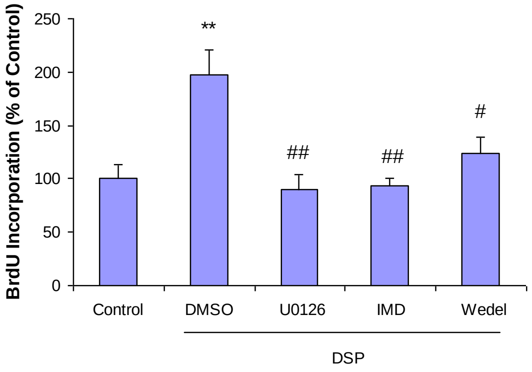

**C**

**FIGURE 1.** DSPs promote cell proliferation through activation of ERK1/2 and NF-κB in human VSMCs. Cultured HASMCs were starved for 24 h in serum-free medium and then different concentrations of DSPs were added for 48 h. 10 µM of PD98059, 10 µM of U0126, 10 µM of SB203580, 5 µM of wedelolactone, 30 nM of IMD-0354, 1 µM of dexamethasone, 10 µM of mecamylamine, 1 µM of α-bungarotoxin, and 10 or 30 µg/ml of polymyxin B were added to cells 30 min prior to treatment with 0.2 µl/ml of DSPs. (A) Bar graphs show concentration-dependent cell proliferation effect of DSPs (from 0.05 to 0.2 µl/ml) as determined by cell proliferation assay; 20 nM of ET-1 was added to cells after 30 min of treatment with DSPs. (B) Bar graphs show the effects of inhibitors on DSP-induced cell proliferation as determined by cell proliferation assay. (C) Bar graphs show the effects of inhibitors on DSP-induced BrdU incorporation. Data represent mean ± S.E.M. ***p* < 0.01, ****p* < 0.001 vs. control; ###*p* < 0.001 vs. control of culture with ET-1 (A); #*p* < 0.05, ##*p* < 0.01, ###*p* < 0.001 vs. DMSO (B,C); &*p* < 0.05, &&*p* < 0.01, &&&*p* < 0.001; PD = PD98059; SB = SB203580; Wedel = wedelolactone; IMD = IMD-0354; Dexa = dexamethasone; Mecam = mecamylamine; α-Bunga = α-bungarotoxin; PMB = polymyxin B.

(Fig. 2A). As verified by western blot, the activation of ERK1/2 was found after 1-h stimulation with DSPs, declined at 2 h, and returned to the baseline at 3 h (Fig. 2B). The activation of NF-κB was elicited after 2-h stimulation with DSPs and remained at this level at 3 h (Fig. 2C). Elk-1, a member of the ETS oncogene family of transcription factors and a downstream transcription factor activated by phosphorylated ERK1/2, was also observed in human VSMCs. DSPs significantly increased phosphorylated Elk-1 after 24 h of treatment in the VSMC nucleus (Appendix, Fig. 1A).

### DSPs Increase the Expression of CyclinD1 and Cdk4 through Activation of ERK1/2 and NF-κB in HASMCs

Mitogenic stimulation through multiple signaling pathways to induce entry and progression through the G1 phase is dependent on the transcription of immediate early genes, D-type cyclins, and cdk4/6[24]. Nuclear accumulation of cyclinD-dependent kinases initiates the sequential activation of protein complexes for the entry into the cell cycle S phase[24]. We investigated the expression and localization of cyclinD1 and cdk4 by combined use of immunofluorescence and western blot. We observed that DSPs increased the expression of cyclinD1 and cdk4 (Fig. 3). The nuclear accumulation of cyclinD1 and cdk4 was also seen after 24 h of treatment with DSPs by confocal imaging (Fig. 3A,B). DSPs significantly increased the expression of cdk4 in the nuclear area (Fig. 3A). Inhibition of phosphorylated ERK1/2 by U0126 or phosphorylated NF-κB by IMD-0354 attenuated the DSP-induced expression of cyclinD1 and cdk4 (Fig. 3A,C).

### DSPs Increase the Expression of ICAM-1 and the Release of IL-6 through Activation of ERK1/2 and NF-κB in HASMCs

In response to cytokine stimulation, downstream signal molecules are subsequently activated and may lead to cell inflammation, cell proliferation, or cell death. The effects of DSPs on ICAM-1 and inflammatory cytokine IL-6 were measured by immunofluorescence, western blot, or single analyte ELISArray. We found that DSPs increased the expression of ICAM-1 and the release of IL-6 (Fig. 4 and Appendix, Fig. 1B). These effects were inhibited by both MEK and NF-κB inhibitors, but not by the LPS inhibitor polymyxin B (Fig. 4).

### DSPs Reduce Serum Starvation–Induced Apoptosis/Necrosis and Increase the Expression of Bcl-2 through Activation of ERK1/2 and NF-κB in HASMCs

The number of living cells is dependent on the balance between cell proliferation and cell death. To further determine the effect of DSPs on cell death, we investigated the effect of DSPs on enrichment factors of DNA fragmentation in the cytoplasm (apoptosis) and in culture medium (necrosis) by using Cell Death Detection ELISA. Compared with the negative control cells, which were cultured with complete serum, serum starvation caused apoptosis and necrosis (Fig. 5A,B), although the obvious cell death was not able to be seen in serum-starved cells. The cell death evoked by serum starvation was reduced by 48 h of treatment with DSPs, and this effect was inhibited by pretreatment with either MEK1/2 inhibitor U0126 or NF-κB inhibitors IMD-0354 and wedelolactone (Fig. 5A,B). To understand the possible mechanisms involved in DSP-induced anti–cell death in serum-starved HASMCs, we detected the expression of Bcl-2, an antiapoptosis protein, by immunofluorecence. The 24 h of treatment with DSPs caused an increase in the expression of Bcl-2 and this effect was inhibited by both U0126 and IMD-0354 (Fig. 5C). Interestingly, the distribution of Bcl-2 protein expression in cells was changed after cells were exposed to DSPs. The expression of Bcl-2 was predominately seen in the cytoplasm by confocal imaging (Fig. 5C).

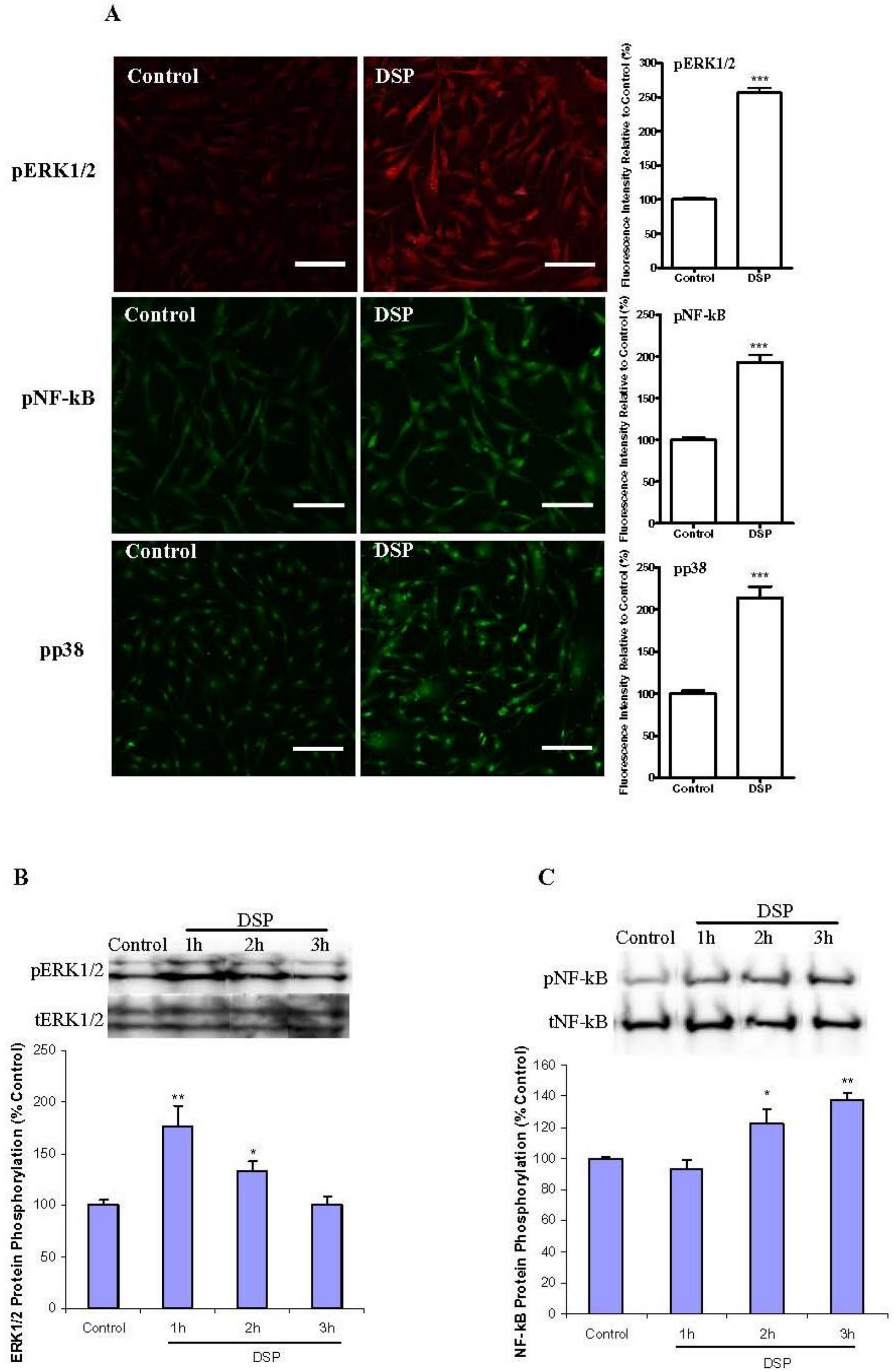

**FIGURE 2.** DSPs enhance phosphorylation of ERK1/2, NF-κB, p38, and Elk-1 in human VSMCs. Serum-starved cells were treated with 0.2 µl/ml of DSPs for 1, 2, or 3 h. (A) Bar graphs show the results of statistical analysis of the fluorescence intensities for phosphorylated ERK1/2, NF-κB, and p38 from samples treated with DSPs for 2 h. The left panels indicate representative images of immunofluorescence. The scale bar in each image represents 20 µm. (B and C) Bar graphs show DSP-induced time-dependent activation of ERK1/2 (B) and NF-κB (C). The upper panels indicate representative autoradiographs of western blot. Data represent mean ± S.E.M. *$p < 0.05$, **$p < 0.01$, ***$p < 0.001$ vs. control; p = phosphorylated; t = total.

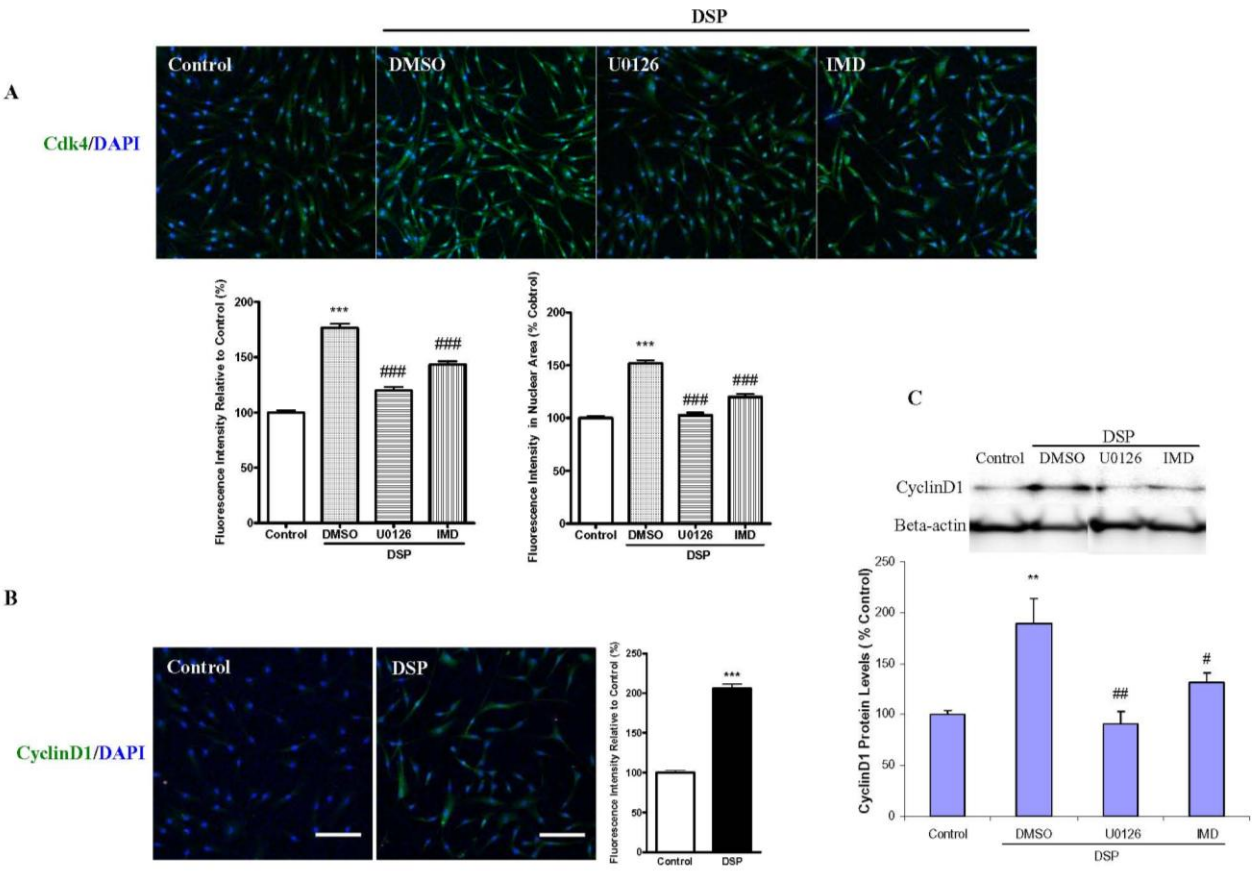

**FIGURE 3.** DSPs increase cyclinD1 and cdk4 expression through activation of ERK and NF-κB in human VSMCs. Serum-starved cells were treated with 0.2 µl/ml of DSPs for 24 h. 10 µM of U0126 or 30 nM of IMD-0354 were added to cells 30 min prior to treatment with DSPs. (A) Bar graphs show the effects of inhibitors on DSP-induced expression of cdk4 in whole cell (the left graph) and in nuclear area (the right graph). The upper panels indicate representative images of immunofluorescence for cdk4 (green) and nuclear DAPI staining (blue). The scale bar in each image represents 20 µm. (B) Bar graphs show the results of statistical analysis of the fluorescence intensities for the expression of cyclinD1. The left panels indicate representative images of immunofluorescence for cyclinD1 (green) and nuclear DAPI staining (blue). The scale bar in each image represents 20 µm. (C) Bar graphs show the effects of inhibitors on DSP-induced expression of cyclinD1. The upper panels indicate representative autoradiographs of western blot. Data represent mean ± S.E.M. $^{**}p < 0.01$, $^{***}p < 0.001$ vs. control; $\#p < 0.05$, $\#\#p < 0.01$, $\#\#\#p$ $<0.001$ vs. DMSO; IMD = IMD-0354.

### Interaction of DSP-Induced Phosphorylation of ERK1/2 and NF-κB in HASMCs

The phosphorylation of ERK1/2 and NF-κB found in our studies was involved in DSP-induced cell proliferation and anti–cell death. To understand the possible cross-talk between two signal molecules, we investigated the effects of their inhibitors on phosphorylation of both ERK1/2 and NF-κB by phosphoELISA assay. DSP-induced phosphorylated ERK1/2 was inhibited by both U0126 and wedelolactone (Fig. 6A), and DSP-induced phosphorylation of NF-κB was blocked by both MEK1/2 inhibitor U0126 and NF-κB inhibitors IMD-0534 and wedelolactone (Fig. 6B).

### DISCUSSION

Epidemiological studies have shown that there is a clear association between cigarette consumption, second hand smoke (SHS) exposure, and cardiovascular diseases[25]. However, the underlying molecular mechanisms to explain how cigarette smoke exposure results in cardiovascular disease are still elusive. Our

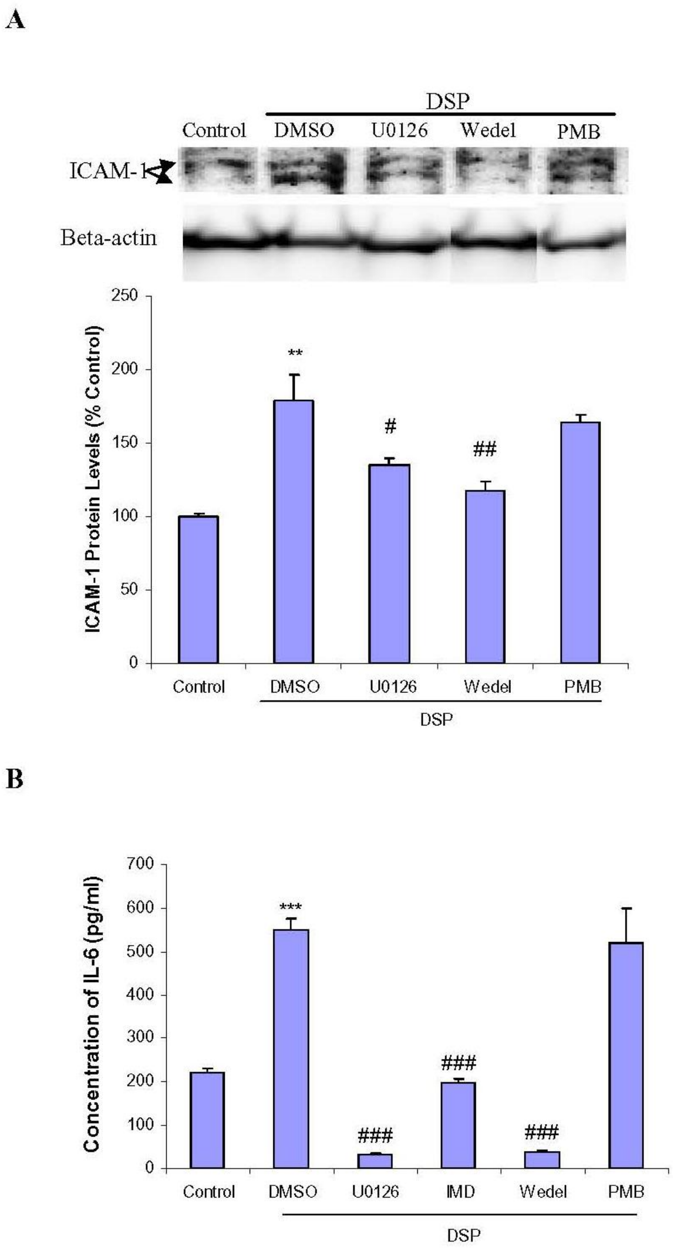

**FIGURE 4.** DSPs induce release of IL-6 and expression of ICAM-1 through activation of ERK1/2 and NF-κB in human VSMCs. Serum-starved cells were treated with 0.2 µl/ml of DSPs for 24 h. 10 µM of U0126, 5 µM of wedelolactone, 30 nM of IMD-0354, or 30 µg/ml polymyxin B were added to cells 30 min prior to treatment with DSPs. (A) Bar graphs show the effects of inhibitors on DSP-induced expression of ICAM-1. The upper panels indicate representative autoradiographs of western blot. (B) Bar graphs show the effects of inhibitors on DSP-induced release of IL-6 as determined by ELISA. Data represent mean ± S.E.M. **$p < 0.01$, ***$p < 0.001$ vs. control; #$p < 0.05$, ##$p < 0.01$, ###$p < 0.001$ vs. DMSO; Wedel = wedelolactone; IMD = IMD-0354; PMB = polymyxin B.

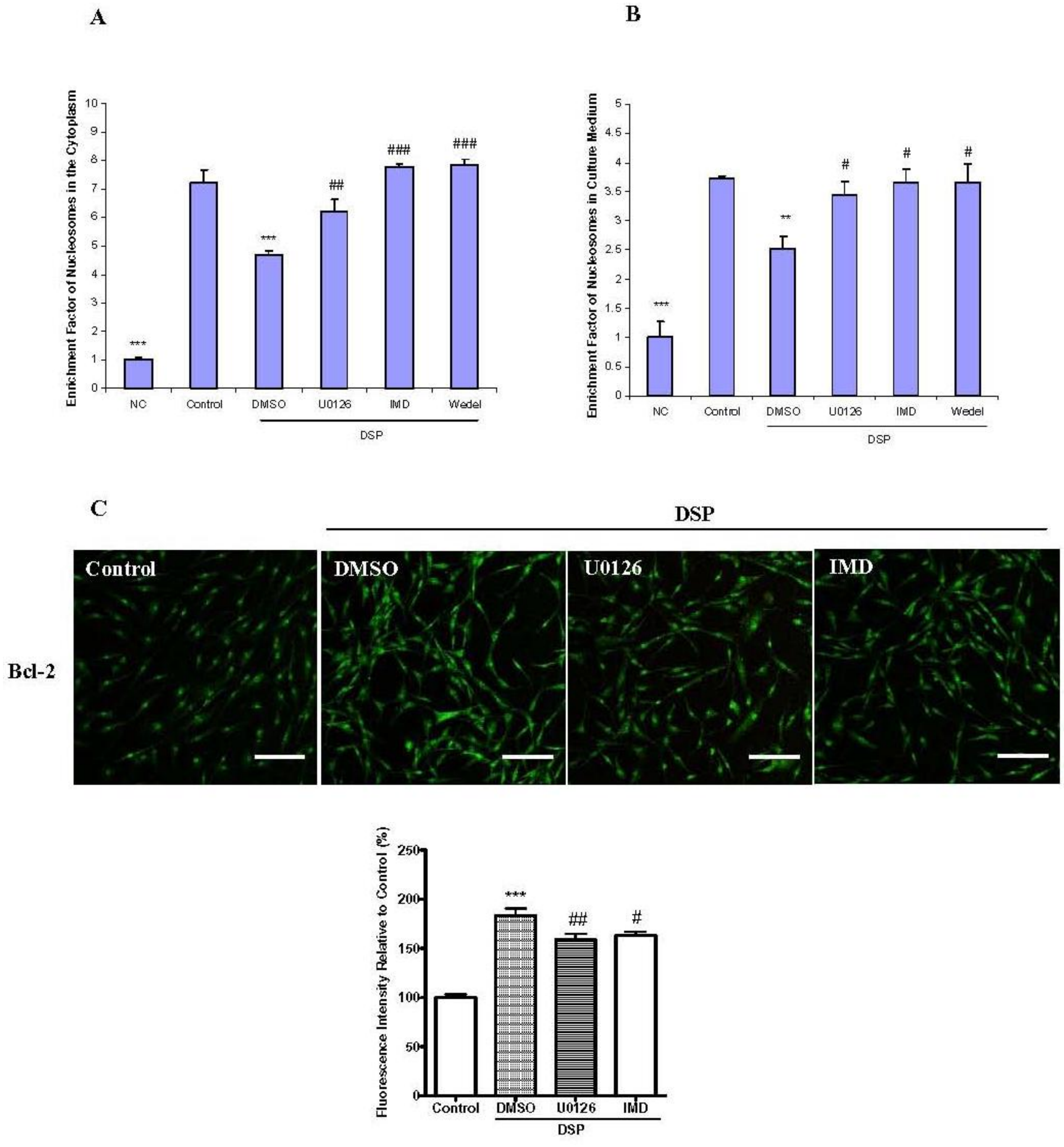

**FIGURE 5.** DSPs reduce serum starvation–induced apoptosis/necrosis and increase expression of Bcl-2 through activation of ERK and NF-κB in human VSMCs. Serum-starved cells were treated with 0.2 μl/ml of DSPs for 24 (C) or 48 h (A,B). The cells cultured in complete medium were regarded as negative control (NC). 10 μM of U0126, 5 μM of wedelolactone, or 30 nM of IMD-0354 were added to cells 30 min prior to treatment with DSPs. (A) DSP-induced anti–cell apoptosis is inhibited by MEK or NF-κB inhibitors. The rate of apoptosis is reflected by the enrichment factor of nucleosomes in the cytoplasm as determined by Cell Death Detection ELISA. (B) DSP-induced anti–cell necrosis is inhibited by MEK or NF-κB inhibitors. The rate of necrosis is reflected by the enrichment factor of nucleosomes in culture medium as determined by Cell Death Detection ELISA. (C) Bar graphs show the effects of MEK and NF-κB inhibitors on DSP-induced expression of Bcl-2. The upper panels indicate representative images of immunofluorescence. The scale bar in each image represents 20 μm. Data represent mean ± S.E.M. **$p < 0.01$, ***$p < 0.001$ vs. control; #$p < 0.05$, ##$p < 0.01$, ###$p < 0.001$ vs. DMSO; Wedel = wedelolactone; IMD = IMD-0354.

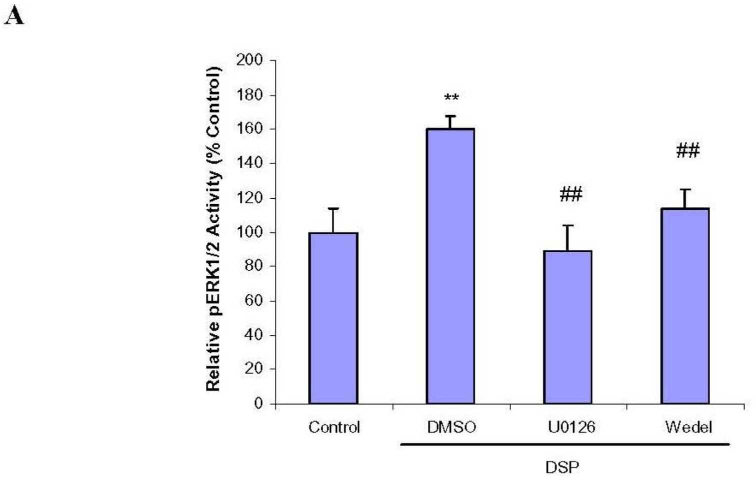

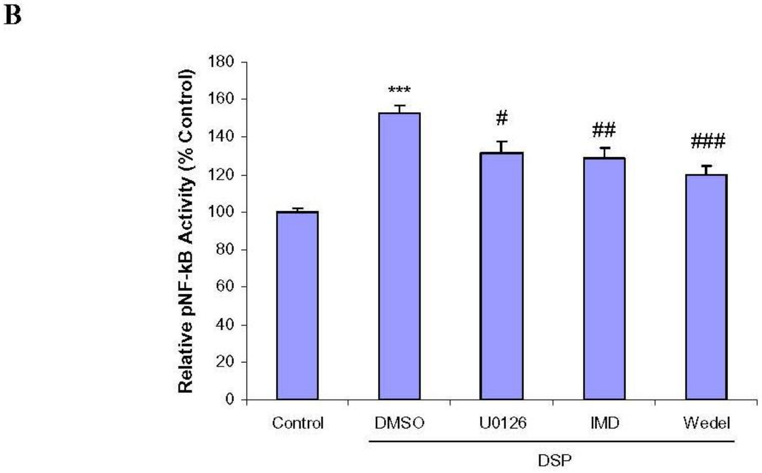

**FIGURE 6.** Interaction of the activation of ERK and NF-κB in DSP-treated human VSMCs. Serum-starved cells were treated with 0.2 µl/ml of DSPs for 1 or 3 h. 10 µM of U0126, 5 µM of wedelolactone, or 30 nM of IMD-0354 were added to cells 30 min prior to treatment with DSPs. Bar graphs show the effects of inhibitors on DSP-induced phosphorylation of ERK1/2 (A) and phosphorylation of NF-κB (B) as determined by phosphoELISA. Data represent mean ± S.E.M. ***p* < 0.01, ****p* < 0.001 vs. control; #*p* < 0.05, ##*p* < 0.01, ###*p* < 0.001 vs. DMSO; p = phosphorylated; Wedel = wedelolactone; IMD = IMD-0354.

previous studies showed that both lipid- and water-soluble smoke extracts promoted cell proliferation in HASMCs, but the effect of lipid-soluble smoke extracts occurred earlier. Furthermore, lipid-soluble, but not water-soluble smoke extracts increased the GPCR-mediated contractile responses in rat vasculature and airways[21]. DSPs include both lipid- and water-soluble particles from cigarette smoke. The present study demonstrates that DSPs induce an inflammatory response, stimulate cell proliferation, and reduce cell death through ERK1/2- and NF-κB–dependent signal pathways in human VSMCs. Cell proliferation is induced by mitogenic stimulation through multiple signaling pathways. Entry into the S phase of the cell cycle is regulated by sequential activation of protein complexes, including D-type cyclins and cdk4/6 in the early G1 phase, followed by the activation of cyclinE-cdk2 complexes in the late G1 phase for progression towards the S phase[24]. The mitogen-dependent accumulation of cyclinD-dependent kinases initiates the phosphorylation of the retinoblastoma protein (Rb). Rb represses the transcription of genes whose products are required for DNA synthesis. However, Rb phosphorylation releases transcription factors, such as the E2F family, thereby promoting expression of target genes necessary for progression towards the S phase. Apart from genes that regulate DNA metabolism, E2Fs induce additional phosphorylation by the cyclinE-cdk2 complex. The mitogen-independent cyclinE-cdk2 collaborates with the cyclinD-dependent kinases to complete Rb phosphorylation. DSP-induced cell proliferation occurred in parallel with increased expression of cyclinD1-cdk4, but not cyclinE-cdk2 (data not shown), indicating that DSP-induced cell proliferation is dependent on transcription of cyclinD1-cdk4 in the early G1 phase of HASMCs.

Mitogenesis is initiated and regulated through multiple signaling pathways. The MAPK-ERK1/2 signaling pathway is responsible for cell proliferation and differentiation, while the MAPK-p38 and MAPK-c-Jun $NH_2$-terminal kinase (JNK) signaling pathways are mainly involved in cell apoptosis and inflammation[26]. The involvement of NF-κB in the control of proliferation is controversial[17,27,28,29]. In the present study, we observed activation of ERK1/2, NF-κB, and p38 in HASMCs after exposure to DSPs. Phosphorylated ERK1/2 increased at 1 h and returned to the baseline at 3 h; the activation of NF-κB was seen later during the time course study of exposure of HASMC to DSPs. DSP-induced activation of ERK1/2 and NF-κB was not found within 30 min (data not shown). MEK1/2 (U0126) or NF-κB (wedelolactone or IMD-0354) inhibitors prevented DSP-induced cell proliferation and cyclinD1-cdk4 expression, whereas p38 inhibitor SB203580 had no effect. This indicates that the cell signal response to DSPs occurs via activation of ERK1/2 and NF-κB, but not p38. Apart from the inhibition of NF-κB and AP-1, dexamethasone is reported to inhibit post-translational modification of gene expression, JNK and p38 MAPKs, and histone deacetylase[30,31,32]. AP-1 is a transcription factor that can be activated upstream by three groups of MAPK signal pathways (ERK1/2, p38, and JNK) and involved in a variety of cellular responses[33]. With 1 μM of dexamethasone, we did not find an improved cell proliferation inhibitory effect compared with NF-κB inhibitors or the MEK1/2 inhibitor. This suggests that the MAPK-JNK pathway and post-translational modification of gene expression may not be involved in DSP-induced cell proliferation. It has been reported that LPS (bacterial endotoxin) is an active component of cigarette smoke[34]. In the present study, the natural peptide polymyxin B was recruited as an inhibitor of LPS[35]. The result indicates that LPS-dependent downstream signal transduction, such as the Toll-like receptor 4 (TLR4)-NF-κB pathway[36] is not responsible for DSP effects. Pestana et al. have shown that nicotine administration at 1 μM (162.2 ng/ml) stimulated mitogenesis in human VSMCs[37]. The plasma concentration of nicotine varies between 4 and 72 ng/ml, and the average concentration is 33 ng/ml in smokers[38]. In the present study, the standard nicotine content in stock DSP solution is 0.1 mg/ml[22], and nicotine content is only 5 or 20 ng/ml when exposed to 0.05 or 0.2 μl/ml of DSPs. 5 and 20 ng/ml of nicotine had no effect on cell proliferation (data not shown) and nicotinic AChR blockers (mecamylamine and α-bungarotoxin) could not inhibit DSP-induced cell proliferation. Hence, nicotine is not responsible for the DSP effects, which also agrees well with previous studies[11,39].

Cell death includes two distinct processes leading to cell death: cell apoptosis and cell necrosis. Serum starvation results in cell growth arrest and keeps cells under a condition of low-level metabolism. We observed both cell apoptosis and cell necrosis of HASMCs induced by serum starvation. Microscopy did not reveal any obvious cell death after HASMC starvation for 3 days. However, using the more sensitive Cell Death Detection ELISA, both apoptosis and cell necrosis were detected in the serum-starved cells. When HASMC cultures were exposed to 0.2 μl/ml of DSPs for 48 h, cell death was reduced. At higher concentration of DSPs (0.8 μl/ml), an obvious cell death occurred in HASMC cultures (data not shown). This implies that a high concentration of DSPs exerts direct toxic effects. Bcl-2, a 26-kDa antiapoptosis protein, inhibits apoptosis by localizing the expression in the endoplasmic reticulum or mitochondria[40,41,42,43]. The studies reveal that NF-κB suppresses apoptosis in a variety of cells[15,16,17,18,19,20]. In the present study, DSPs inhibited cell death and increased the expression of Bcl-2, both of which were prevented by either MEK or NF-κB inhibitors. This indicates that DSP-induced inhibition of cell death occurs through activation of both ERK1/2- and NF-κB–dependent pathways.

The pathogenesis of atherosclerosis is today considered to involve excessive and chronic inflammation in the arterial wall[4,5,6]. It has been reported that cigarette smoke exposure activates the nicotinamide adenine dinucleotide phosphate (NADPH) oxidase–dependent NF-κB signal transduction system, leading to proinflammatory alterations in rat vascular phenotype[44]. In the present study, we examined the effects of DSPs on synthesis and release of IL-6 and expression of ICAM-1 in HASMCs. Both MEK and NF-κB inhibitors attenuated the DSP-induced increase in the level of IL-6 and the expression of ICAM-1, but the LPS inhibitor polymyxin B had no effect. This suggests that the DSP-induced inflammatory response depends on ERK1/2- and NF-κB–mediated inflammatory signalings, whereas LPS-dependent signal transduction may not be involved in it.

ERK1/2 activation is generally reported to require sequential activation of small G protein and MEK[45]. Protein kinase C, protein kinase A, and phosphoinositide 3-kinase are thought to act upstream from MAPK-ERKs mediated by GPCRs[23,46]. Activated ERK1/2 translocates into the nucleus and subsequently phosphorylates different transcription factors, such as Elk-1, and then modulates the expression of genes responsible for cell proliferation, antiapoptosis, and inflammation[47,48,49]. Activation of ERK1/2 may also directly or indirectly lead to phosphorylation of NF-κB[50,51,52]. Here we demonstrate that the MEK inhibitor can abolish DSP-induced activation of NF-κB, and the NF-κB inhibitor can attenuate DSP-induced phosphorylation of ERK1/2, suggesting an interaction between activation of ERK1/2, Elk-1, and NF-κB.

In conclusion, the present study has demonstrated that lipid-soluble smoke particles (1) promote HASMC cell proliferation, (2) inhibit cell apoptosis/necrosis, and (3) induce an inflammatory response in HASMCs. MEK or NF-κB inhibitors attenuate the DSP effects, suggesting that both intracellular ERK1/2 and NF-κB signal pathways are involved (Appendix, Fig. 2). Exploration of the intracellular signal transduction mechanisms behind cigarette smoke effects may lead to new options for the treatment of cardiovascular diseases associated with smoking.

### AUTHOR´S CONTRIBUTIONS

QC carried out the main part of the experiments, participated in the design, statistical analysis, drafting, and writing of the manuscript. LE participated in the writing of the manuscript. CX conceived the study and the design, coordinated the work and the writing of the manuscript. All authors have read and approved the final manuscript.

### ACKNOWLEDGMENTS

This study was supported by the Swedish Heart-Lung Foundation, Swedish Research Council (Grant No. 5958), Sweden, and the Flight Attendant Medical Research Institute (FAMRI, USA).

## APPENDIX

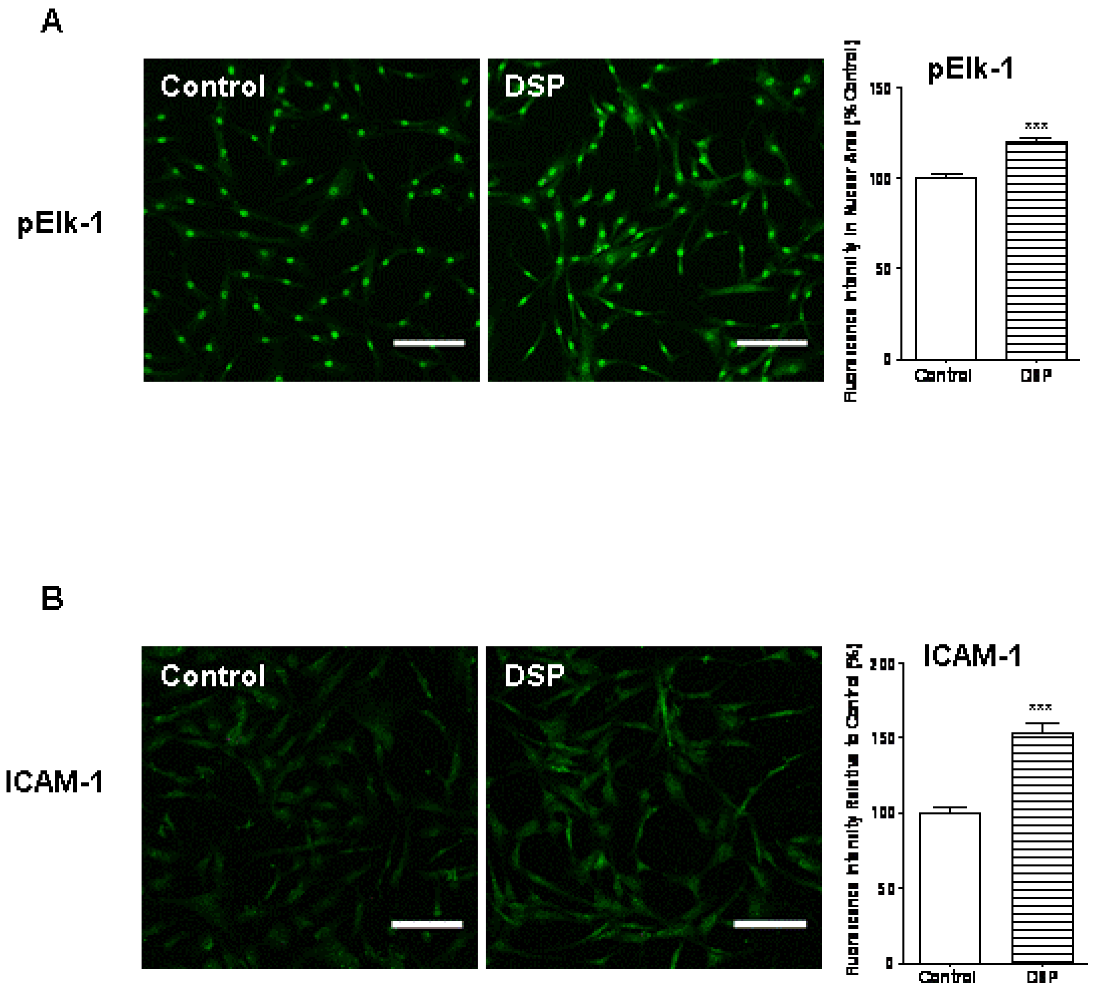

**FIGURE 1.** The data provided represent the immunofluorescence analysis of DSP-induced expression of ICAM-1 and phosphorylation of Elk-1. Serum-starved cells were treated with 0.2 µl/ml of DSPs for 24 h. Bar graphs show the results of statistical analysis of the fluorescence intensities for phosphorylated Elk-1 in nuclear area (A) and the expression of ICAM-1 (B). The left panels indicate representative images of immunofluorescence. The scale bar in each image represents 20 µm. Data represent mean ± S.E.M. ***$p < 0.001$ vs. control; p = phosphorylated.

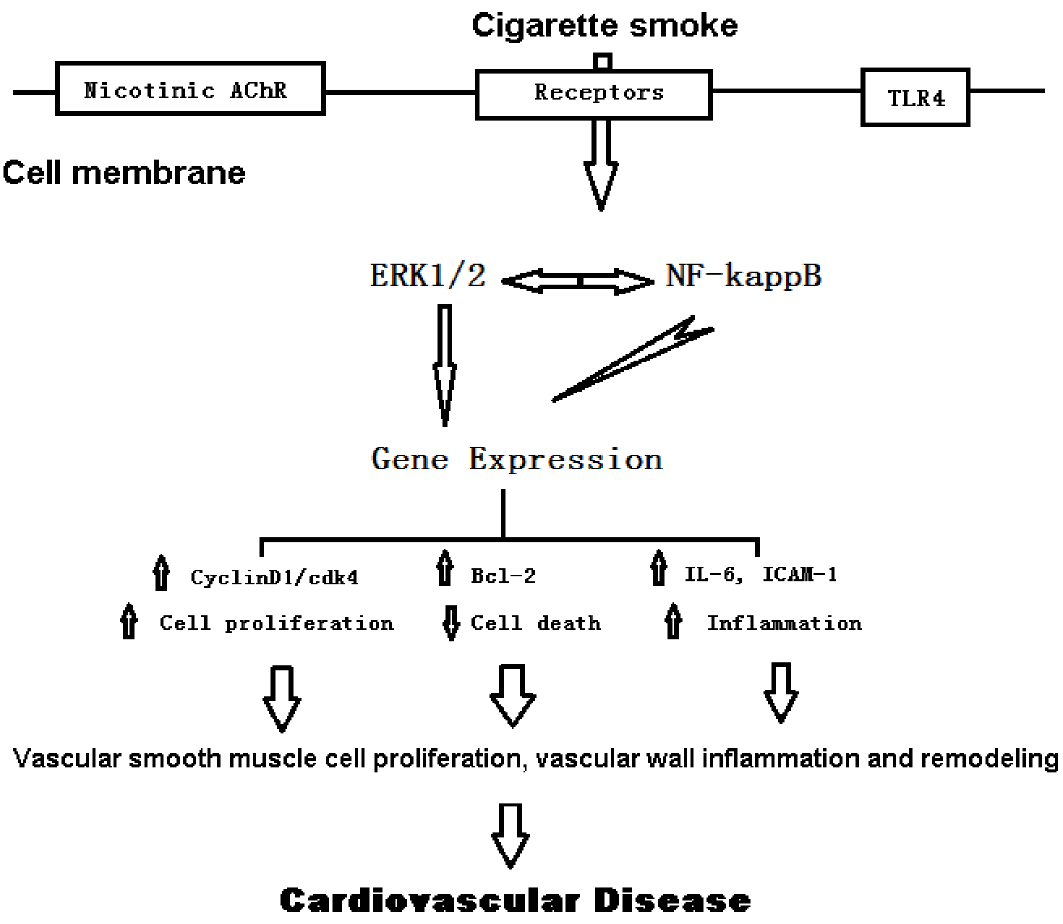

**FIGURE 2.** Schematic diagram of the pathways activated by cigarette smoke in human VSMCs. The diagram shows the signal pathways that may involve DSP-induced cell proliferation, anti–cell death, and inflammatory response in HASMCs.
